# New insights into pediatric idiopathic pulmonary hemosiderosis: the French RespiRare® cohort

**DOI:** 10.1186/1750-1172-8-161

**Published:** 2013-10-14

**Authors:** Jessica Taytard, Nadia Nathan, Jacques de Blic, Mickael Fayon, Ralph Epaud, Antoine Deschildre, Françoise Troussier, Marc Lubrano, Raphaël Chiron, Philippe Reix, Pierrick Cros, Malika Mahloul, Delphine Michon, Annick Clement, Harriet Corvol

**Affiliations:** 1 Pediatric Pulmonary Department, AP-HP, Hôpital Trousseau, Université Pierre et Marie Curie-Paris6, Inserm U938, 26, avenue du Docteur Arnold-Netter, 75012 Paris, France; 2 Pediatric Pulmonary Department, AP-HP, Hôpital Necker Enfants Malades, Paris, France; 3 CHU de Bordeaux, Centre d’Investigation Clinique (CIC 0005), F-33076 Bordeaux, France; 4 Pediatric Department, Centre Hospitalier Inter-communal de Créteil, Créteil, France; 5 Pediatric Department, Centre Hospitalier Universitaire de Lille, Lille, France; 6 Pediatric Department, Centre Hospitalier Universitaire d’Angers, Angers, France; 7 Pediatric Department, Centre Hospitalier Universitaire de Rouen, Rouen, France; 8 Pediatric Department, Centre Hospitalier Universitaire de Montpellier, Montpellier, France; 9 Pediatric Pulmonary Department, Centre Hospitalier Universitaire de Lyon, Lyon, France; 10 Pediatric Department, Centre Hospitalier Universitaire de Brest, Brest, France

**Keywords:** Idiopathic pulmonary hemosiderosis, Child, Interstitial lung disease, Autoimmune lung disease, Coeliac disease, Down syndrome

## Abstract

**Background:**

Idiopathic pulmonary hemosiderosis (IPH) is a rare cause of alveolar hemorrhage in children and its pathophysiology remains obscure. Classically, diagnosis is based on a triad including hemoptysis, diffuse parenchymal infiltrates on chest X-rays, and iron-deficiency anemia. We present the French pediatric cohort of IPH collected through the French Reference Center for Rare Lung Diseases (RespiRare®, http://www.respirare.fr).

**Methods:**

Since 2008, a national network/web-linked RespiRare® database has been set up in 12 French pediatric respiratory centres. It is structured as a medical recording tool with extended disease-specific datasets containing clinical information relevant to all forms of rare lung diseases including IPH.

**Results:**

We identified 25 reported cases of IPH in children from the database (20 females and 5 males). Among them, 5 presented with Down syndrome. Upon diagnosis, median age was 4.3 [0.8-14.0] yrs, and the main manifestations were: dyspnea (n = 17, 68%), anemia (n = 16, 64%), cough (n = 12, 48%), febrile pneumonia (n = 11, 44%) and hemoptysis (n = 11, 44%). Half of the patients demonstrated diffuse parenchymal infiltrates on chest imaging, and diagnosis was ascertained either by broncho-alveolar lavage indicating the presence of hemosiderin-laden macrophages (19/25 cases), or lung biopsy (6/25). In screened patients, initial auto-immune screening revealed positive antineutrophilic cytoplasmic antibodies (ANCA) (n = 6, 40%), antinuclear antibodies (ANA) (n = 5, 45%) and specific coeliac disease antibodies (n = 4, 28%). All the patients were initially treated by corticosteroids. In 13 cases, immunosuppressants were introduced due to corticoresistance and/or major side effects. Median length of follow-up was 5.5 yrs, with a satisfactory respiratory outcome in 23/25 patients. One patient developed severe pulmonary fibrosis, and another with Down syndrome died as a result of severe pulmonary hemorrhage.

**Conclusion:**

The present cohort provides substantial information on clinical expression and outcomes of pediatric IPH. Analysis of potential contributors supports a role of auto-immunity in disease development and highlights the importance of genetic factors.

## Introduction

Idiopathic pulmonary hemosiderosis (IPH) is a rare cause of alveolar hemorrhage of unknown etiology in children, leading to chronic infiltrative pulmonary disease [1-4]. It is classically characterized by a triad of hemoptysis, iron-deficiency anemia and pulmonary infiltrates on chest X-rays; and usually occurs before the age of 10 years (yrs) old [5-10]. The diagnosis of IPH is confirmed by bronchoscopy with bronchoalveolar lavage (BAL) showing hemosiderin-laden macrophages called siderophages, pathognomonic of the disease [11]. Lung biopsies are sometimes required to eliminate differential diagnoses such as vasculitis [12]. First line treatments usually include systemic corticosteroid therapy. This may be substituted by immunosuppressants in case of corticosteroid resistance or dependence and/or unfavorable outcome [2,3,13,14].

Various hypotheses have been proposed to explain the pathophysiology of IPH: allergic, environmental, genetic and/or auto-immune. The allergic hypothesis is based on the frequent association between IPH and cow’s milk allergy [15]. The environmental theory has been suggested after the emergence of IPH in children exposed to *Stachybotris chartarum*[16,17]. IPH has also been described in siblings, leading to discuss a genetic theory, but no gene has yet been identified [18,19]. Finally, the auto-immune theory is recognized as the most probable, considering the frequent association with auto-immune diseases such as coeliac disease, glomerulonephritis and/or rheumatoid arthritis [2,5,11,17,20-23].

Pediatric IPH is a rare respiratory disease whose incidence varies between 0.24 and 1.26 per million [3]. This explains the limited knowledge on physiopathology and the lack of consensus regarding care. Through the organization of the French Reference Center for Rare Lung Diseases (RespiRare®) with the national network/web-linked database (described previously), we reviewed the IPH cases in the French pediatric population with two main goals: to update management strategies and to provide new insights into disease contributors and physiopathology [24].

## Material and methods

Since 2008, the RespiRare® database has been set up in 12 French pediatric respiratory centres. This database is structured as a medical recording tool for the patient, with extended disease-specific datasets containing clinical information relevant to all kinds of rare lung diseases including interstitial lung disease and IPH [24]. The database and the data collection have been approved by French national data protection authorities [24]. Each patient and/or his legal representatives are informed prior to entering their data into the database. This study is a chart based retrospective study. Relevant clinical data of the French pediatric IPH patients were retrospectively collected via the RespiRare® database. Diagnosis is based on the triad of iron deficiency anemia, respiratory symptoms (including dyspnea, cough and hemoptysis), and pulmonary infiltrates on chest imaging, and is confirmed by the presence of hemosiderin laden macrophages in the BAL and/or on lung tissue specimens.

At diagnosis, the data collected comprised:

(i) clinical features: age, body mass index (BMI), hemoptysis, dypnea, cough and/or febrile pneumonia; personal and/or familial history of allergy and/or auto-immune diseases;

(ii) imaging data: chest X-ray and chest CT scan;

(iii) respiratory function tests: spirometry measurements (vital capacity (VC), forced vital capacity (FVC), slow vital capacity (SVC) and forced expiratory volume in one second (FEV_1_)) and single-breath measure of carbon monoxide diffusion (DLCO);

(iv) BAL: the percentage of hemosiderin laden macrophages, and the Golde score. The Golde score is a semi-quantitative method for assessing the hemosiderin content of alveolar macrophages after Prussian blue stain: at least 200 alveolar macrophages are examined and, to each macrophage, is attributed a score ranging from 0 (absence of blue staining) to 4 (hemosiderin laden macrophages); the Golde score represents the mean score on 100 macrophages (that could range from 0 to 400). An alveolar hemorrhage is confirmed for a score higher than 20, and characterized as severe for a score above 100 [20];

(v) lung biopsy;

(vi) blood tests: hemoglobin, reticulocytes count, and autoimmune assessments: antitransglutaminase (ATA, IgA and IgG), antigliadin (AGA) and antiendomysium (AEA) antibodies, antinuclear antibodies (ANA), anti-double strand DNA and anti-smooth-muscle antibodies (SMA), rheumatoid factor (RF) and antineutrophilic cytoplasmic antibodies (ANCA).

The data collected during the follow up included treatments, side effects and clinical outcome.

Descriptive data analysis (medians and standard deviations for quantitative values, percentages for qualitative values) have been realised using the Excel 2010 software.

## Results

### Population demographic description at diagnosis

Twenty-five patients (20 girls and 5 boys), diagnosed with IPH between 1996 and June 2012, have been included from 12 French pediatric respiratory centers. The main clinical data at diagnosis are described in Table 1.

**Table 1:** Main manifestations at diagnosis

	**Patients**	**Patients (%)**
Hemoptysis	11	44
Dyspnea	17	68
Cough	12	48
Febrile pneumonia	11	44
Anemia	16	64
Severe anemia (<7 g/dl)	9	36

Median age at diagnosis was 4.3 [0.8-14.0] yrs. Diagnosis was made before the age of 2 yrs in 7 patients, and after the age of 10 yrs in 4 of them. Median BMI was 14.75 [13.3-25.5] with a median Z-score of −0.6 [−2.46-2.16]. The main manifestations at diagnosis were: dyspnea (n = 17, 68%), cough (n = 12, 48%), febrile pneumonia (n = 11, 44%) and hemoptysis (n = 11, 44%). Five patients out of the 25 IPH cases had a concomitant Down syndrome (20%).

Among the 25 patients, 2 had a personal history of coeliac disease and one of arthralgia. Five familial cases of autoimmune diseases were observed: 2 ankylosing polyarthritis, 1 coeliac disease, 1 telangiectasis, 1 type 1 diabetes, and one Minkowski-Chauffard disease. Furthermore, 2 patients were dizygote twins and another patient had 2 cousins with an IPH history.

### Investigations at diagnosis

#### Blood count

Anemia was present in 64% of cases, with a median hemoglobin of 7.2 g/dl [2.3-12.4]. Nine patients had a severe anemia (<7 g/dl). Reticulocytes, documented in only 4 out of the 25 patients, were systematically elevated, varying between 98,900 and 152,150/mm3.

#### Thoracic imaging

**Figure 1:**
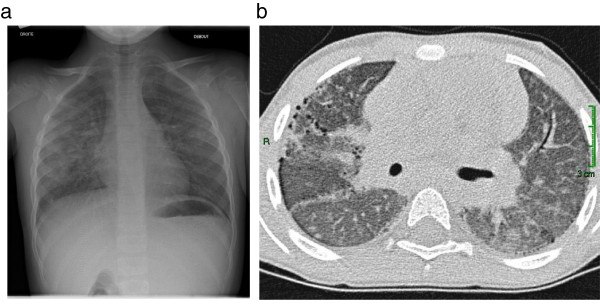
**Thoracic imaging. a** shows ground-glass opacities on a chest X-ray in a 5 yrs old IPH patient. **b** shows on a chest CT-scan an interstitial pattern with micronodules and ground-glass opacities in a 5 yrs old IPH patient.

Interstitial and/or alveolar interstitial patterns were visualized by chest X-rays for 13 patients and by chest CT-scans for 17 patients. The chest CT scans showed ground-glass opacities (12 patients), sub pleural cysts (6 patients), and, less frequently, micronodules (2 patients), thickened interlobular septa (2 patients), honeycomb patterns (1 patient), and traction bronchiectasis (1 patient). Images were often diffuse and bilateral. An illustration is given in Figure 1.

#### Respiratory function tests

Spirometric analyses at onset were available for 18 patients. The 7 remaining patients had less than 3 yrs old at the time of diagnosis, and, thus, did not perform functional respiratory tests. The spirometric analyses were normal in 9/18 patients (50%), showed a restrictive pattern for 6 patients (37.5%) and an airway obstruction for 1 patient (6%). Medians of VC, FVC and SVC were respectively 69, 70 and 65%. For the patients presenting with a restrictive pattern, the median age was 9 yrs old [3]. DLCO, measured for 9 patients, was either decreased (4/9: 44%), or normal.

#### BAL

The diagnosis was ascertained in 19/25 cases by the BAL that showed an accumulation of hemosiderin-laden macrophages. The Golde score, evaluated in 7 cases indicated a median of 158 [52–356].

#### Pulmonary biopsies

Eight patients underwent a lung biopsy, which documented presence of red blood cells in the alveoli and in the interstitium.

#### Auto immune assessments

Results of the initial auto-immune screenings are described in Table 2. In screened patients, it principally revealed positive ANCA (n = 6, 40%), ANA (n = 5, 45%), ASM (n = 3, 50%), and specific cow’s milk IgE (n = 3, 33%). Specific coeliac disease antibodies were screened in 14 patients (56%), and found to be positive in 4 (28% of the tested patients): 3 patients had positive ATA IgA, and 1 positive AGA.

**Table 2:** Auto-immune assessment at diagnosis

	**Tested (n)**	**Positive (n)**	**Positive (%)**
Coeliac disease Ig	14	4	28
Cow's milk allergy	9	3	33
ANCA	15	6	40
Anti nuclear antibodies	11	5	45
Anti smooth muscle antibodies	6	3	50
Rheumatoid factor	10	2	20
Anti ds DNA antibodies	10	1	10
Anti glomerular basal membrane	9	0	0

Ig: immunoglobulin, ANCA: anti neutrophilic cytoplasmic antibodies, ds: double strand

During the follow-up, additional auto-antibodies were documented: RF (n = 3) and ANA (n = 2). Interestingly, repeated evaluation of the auto-immune status in the 6 positive ANCA patients showed variable evolution: ANCA disappeared for 4 patients at the end of the follow-up, decreased for one patient and remained stable for one patient.

### Treatment and follow up

**Figure 2:**
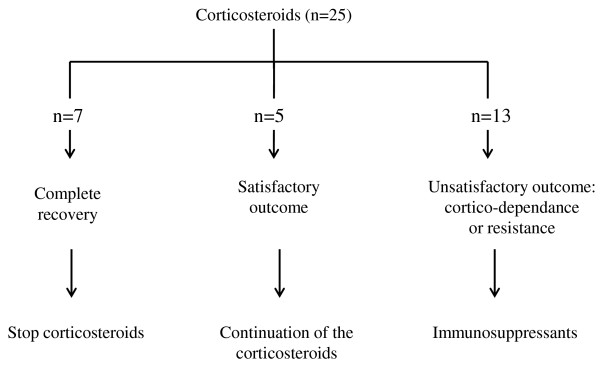
Treatment synopsis.

Treatment synopsis is described Figure 2. As highlighted, all the patients received systemic corticosteroids with a variable efficacy. Intravenous corticosteroids were the usual first line treatment with monthly pulses of methylprednisolone (300 mg/m^2^/day for 3 days per month) for 100% of the patients (n = 25). In situations of severe symptoms, daily oral prednisone was added (1 mg/kg/d). The duration of corticosteroids treatment was variable among the patients based on clinical, radiological (alveolar infiltrates) and biological (hemoglobin, reticulocytes) evolution.

Thirteen patients had an unsatisfactory outcome, including in 6 of them a Cushing syndrome with important gain of weight, stretch marks and hyperthricosis. For these 13 patients, one or more immunosuppressants were added: hydroxychloroquine (4.5 to 6 mg/kg/day for n = 10 patients), mycophenolate mofetil (600 mg/m^2^/day for n = 7 patients), or azathioprine (1 mg/kg/day for n = 1 patient). No side effects were reported, allowing either to interrupt the corticosteroids (n = 6 patients), or to reduce their dosage (n=7 patients).

The median length of follow-up was 5.5 yrs (2 months to 14 yrs). Respiratory outcome was satisfactory in 23 patients, although 7 of them experienced several relapses. The 2 other patients, who were diagnosed at 5 months old, had a severe expression of the disease: 1 of them developed a pulmonary fibrosis, and the other one (with a Down syndrome) had a fatal pulmonary hemorrhage.

## Discussion

Although several pediatric IPH cases have been reported in the literature, few cohorts have been described so far. To our knowledge, this study is one of the largest ever reported, with 25 IPH pediatric cases, and a long median length of follow-up of 5.5 yrs [25].

IPH generally occurs in children below the age of 10 yrs, and commonly between the ages of 1–7 yrs [2,5,8,21]. Although the classical triad of iron deficiency anemia, respiratory symptoms (including dyspnea, cough and hemoptysis), and pulmonary infiltrates on chest imaging is characteristic, clinical presentation is highly variable; anemia and dyspnea being the most frequent clinical features (64% and 68% respectively in our study). Hemoptysis only occurred in 50% of the patients, but its incidence could be underestimated in young children, who frequently swallow their sputum. So far, number of studies highlighted an important delay between onset of symptoms and IPH diagnostic, ranging from 1 to 6.3 yrs [3,7,11,14,21]. In our study, the highest delay reached 16 months. This delay in diagnosis may be due to several factors: insidious onset, and lack of awareness about the condition. Moreover, iron deficiency anemia can also be misleading, especially if respiratory signs are mild.

IPH pathogenesis remains controversial, with, so far, four main etiological hypotheses described in the literature: the environmental, the allergic, the auto-immune, and the genetic hypotheses. In this cohort, we found no evidence favouring an environmental etiology, with, however, little collected information on the various possible environmental factors. This theory was suggested after the occurrence of acute pulmonary hemorrhages in children exposed to *Stachybotris chartarum* in Cleveland*,* but was not further confirmed [16,17]. Similarly, the allergic theory, based on the association with the hypersensitivity to proteins in cow's milk (Heiner syndrome), remains controversial [15]. In the present cohort, 3 patients out of 25 had positive antibodies. As eviction of cow's milk proteins has been shown to benefit the patients with Heiner syndrome, the dosage of cow's milk IgE remains recommended.

Our present study provides support for an auto-immune contribution in IPH physiopathology. First, most of the patients (17 out of the 25) had auto-immune antibodies at onset, and, for 6 patients, additional auto-immune antibodies appeared during the follow-up. The most frequent auto-immune antibodies that were found in our cohort were: SMA (50% of the tested patients); ANA (45%) and ANCA (40%). These antibodies are usually associated with primitive vasculitis and systemic diseases, and rarely reported in IPH patients in the literature [26,27]. Furthermore, several authors described that one out of 4 children with IPH who survive develops an immune disorder [21,22]. In their IPH cohort, Le Clainche *et al.* reported 3 patients out of 15 who displayed rheumatoid arthritis-like symptoms, 6 months to 7 yrs after IPH diagnosis [21]. In our study, the search for RF, performed in 10 patients, gave a positive result in 2 of them. Rheumatoid arthritis is known to be the most frequent systemic disease in the general population (0.5 to 1%) and arthritis is sometimes associated with respiratory symptoms, typically with a diffuse parenchymal lung disease [1]. This would suggest to systematically screen IPH patients for rheumatoid arthritis. Moreover, along with the dosage of RF, we propose to associate the research of anti-citrullinated peptides (anti-CCP). Indeed, several studies have led to suggest that anti-CCPs may be more specific and appear earlier in the course of rheumatoid arthritis than the classical auto-antibodies, even in the absence of clinical manifestations of rheumatoid arthritis or connective tissue disease [28,29]. Of interest, the levels of anti-CCPs were reported to strongly correlate with the variation in DLCO, and possibly to lung disease severity [30].

IPH has frequently been associated with the celiac disease, another auto-immune disorder. This association is well-known as the Lane-Hamilton syndrome, with 14 cases described in the literature [31,32]. In the present study, specific celiac disease anti-bodies (anti-gliadin, anti-endomysium and anti-transglutaminase antibodies) were indeed present in 4 patients among the 14 patients tested (*i.e.* 28% of the tested patients). As a gluten-free diet has been proven beneficial to the evolution of the celiac disease, as well as to the respiratory outcome of the patients with a Lane-Hamilton syndrome, we recommend a systematic screening for celiac disease in IPH patients. Some authors even suggest to systematically perform gastrointestinal endoscopies and biopsies in IPH patients for whom the severity of anemia is disproportionate to radiological findings, even in the absence of gastrointestinal symptoms [7,31]. In addition, it may be worth to include HLA screening to the panel of tests performed in IPH situations. Indeed, HLA DQ2 is present in 90 to 95% of the patients presenting with a celiac disease [33,34].

Information derived from the present cases analysis supports a role of genetic contribution to IPH expression. Two of the patients were dizogote twins, 2 others had relatives with IPH, and 7 had a personal and/or a familial history of auto-immune disorders. An exhaustive research for family history will help developing appropriate genetic studies. We were surprised to find a high prevalence of Down syndromes in our cohort (5 patients out of 25, *i.e.* 20%), the prevalence of the Down syndrome in the French population being 1/2,000 birth per year. To our knowledge, no study had previously reported such an association, which may provide important insights into IPH physiopathology. Indeed, it is known that Down syndrome patients have more frequent autoimmune diseases, with a particular high frequency of celiac disease [35-38]. In our cohort, among the 4 patients tested positive for celiac disease antibodies, 2 had a Down syndrome. Furthermore, we observed that the Down’s syndrome patients with IPH had a worst prognosis compared to the others, including the patient with a fatal outcome, and the 4 others who experienced frequent relapses. One possible explanation may be the higher frequency of lower respiratory tract infections in Down syndrome patients, possibly linked to lower IgG2 serum levels, lower lymphocytes’ count, and/or lower T and NK lymphocytes counts [39].

Several therapeutic approaches have been reported in IPH with various results [3,5,13]. Disease rarity precludes the set-up of randomized controlled trials to evaluate the efficacy of immunomodulatory and/or anti-inflammatory agents for IPH. As indicated in small case series and/or case reports, the main treatments are corticosteroids with various regimens. Corticosteroids have been reported to be associated with decreased pulmonary bleeding relapses and pulmonary fibrosis progression, as well as with higher survival rates [5]. Therapeutic regimens vary across the studies. Immunosuppressive therapies, mainly azathioprine and hydroxychloroquine, are mostly proposed in situations of patients with steroid-refractory disease [3,14,17,21,40,41]. Similar therapeutical strategies were observed in the present cohort, with mainly corticosteroids at diagnosis, then introduction of hydroxychloroquin or mycophenolate mofetil in situations of cortico-dependence and or resistance. In the literature, 2 cases of lung transplantation were reported, but both experienced a recurrence of bleeding within the allograft [40,41].

The major strengths of the present study are the large size of the cohort, with 25 IPH pediatric cases, and the long follow-up, with a median of 5.5 yrs. The largest pediatric cohort published so far was from India, with 26 children with IPH, but with a shorter length of follow-up (mean: 28 ± 27 months) [25]. These strengths have been achieved thanks to the RespiRare® database, which offers a unique opportunity to collect clinical, biological and radiological data from patients with rare respiratory disorders followed in French pediatric respiratory expert centers. This study has, however, limitations, mainly the heterogeneity in investigation protocols and therapeutic regimens. This is a major challenge that will be efficiently addressed with the set-up of the RespiRare® network and the establishment of common procedures of IPH patient management.

To conclude, the review of this large pediatric cohort leads to suggest a probable concomitant auto-immune and genetic etiology of IPH. This implies that a systematic screening for auto-immune diseases should be included in the diagnostic procedure. An important finding is also the high number of IPH patients with Down syndrome, that will need to be further investigated. Furthermore, a structured follow-up including blood tests with reticulocyte counts is critical to improve patient management and to allow an earlier diagnosis and treatment of exacerbations.

## Competing interests

The authors declare that they have no competing interests.

## Authors’ contributions

JT and HC designed the study and drafted the manuscript. All the authors contributed to enter patients’ data in the RespiRare® database and to check their data before publication. NN, AC, JdB and MF provided a special contribution to the data and the manuscript correction. All authors read and approved the final manuscript.
